# 1,1,1-Trichloro-2,2-bis­(4-iodo­phen­yl)ethane

**DOI:** 10.1107/S1600536812032254

**Published:** 2012-07-21

**Authors:** Graham Smith

**Affiliations:** a Science and Engineering Faculty, Queensland University of Technology, GPO Box 2434, Brisbane, Queensland 4001, Australia

## Abstract

In the structure of the title compound, C_14_H_9_Cl_3_I_2_, which is the 4-iodo­phenyl analogue of the insecticide DDT [1,1,1-tri­chloro-2,2-bis­(4-chloro­phen­yl)ethane], isomorphism between the two compounds has been confirmed. In the mol­ecule, the dihedral angle between the planes of the two benzene rings is 65.8 (4)° which compares with 64.7 (7)° in DDT.

## Related literature

For the determination of crystal data for the title compound and the *p*-bromo substituted DDT analogue, see: Schneider & Fankuchen (1946 ▶). For the structures of DDT and related analogues, see: DeLacy & Kennard (1972 ▶); Hovmöller *et al.* (1978 ▶).
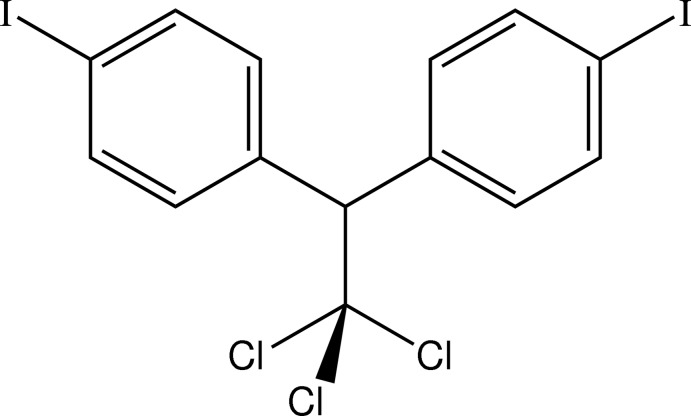


## Experimental

### 

#### Crystal data

C_14_H_9_Cl_3_I_2_
*M* *_r_* = 537.36
Orthorhombic, 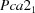

*a* = 9.8117 (3) Å
*b* = 20.3445 (4) Å
*c* = 8.0486 (2) Å
*V* = 1606.61 (7) Å^3^
*Z* = 4
Mo *K*α radiation
μ = 4.40 mm^−1^
*T* = 200 K
0.25 × 0.20 × 0.08 mm

#### Data collection

Oxford Diffraction Gemini-S CCD-detector diffractometer
Absorption correction: multi-scan (*CrysAlis PRO*; Agilent, 2012 ▶) *T*_min_ = 0.386, *T*_max_ = 0.980
5183 measured reflections
2905 independent reflections
2687 reflections with *I* > 2σ(*I*)
*R*_int_ = 0.028

#### Refinement

*R*[*F* ^2^ > 2σ(*F* ^2^)] = 0.044
*wR*(*F* ^2^) = 0.112
*S* = 1.08
2905 reflections
172 parameters
1 restraint
H-atom parameters constrained
Δρ_max_ = 0.80 e Å^−3^
Δρ_min_ = −0.95 e Å^−3^
Absolute structure: Flack (1983 ▶), 1203 Friedel pairs
Flack parameter: −0.02 (4)

### 

Data collection: *CrysAlis PRO* (Agilent, 2012 ▶); cell refinement: *CrysAlis PRO*; data reduction: *CrysAlis PRO*; program(s) used to solve structure: *SIR92* (Altomare *et al.*, 1993 ▶); program(s) used to refine structure: *SHELXL97* (Sheldrick, 2008 ▶) within *WinGX* (Farrugia, 1999 ▶); molecular graphics: *PLATON* (Spek, 2009 ▶); software used to prepare material for publication: *PLATON*.

## Supplementary Material

Crystal structure: contains datablock(s) global, I. DOI: 10.1107/S1600536812032254/su2477sup1.cif

Structure factors: contains datablock(s) I. DOI: 10.1107/S1600536812032254/su2477Isup2.hkl

Supplementary material file. DOI: 10.1107/S1600536812032254/su2477Isup3.cml

Additional supplementary materials:  crystallographic information; 3D view; checkCIF report

## Figures and Tables

**Fig. 1.**
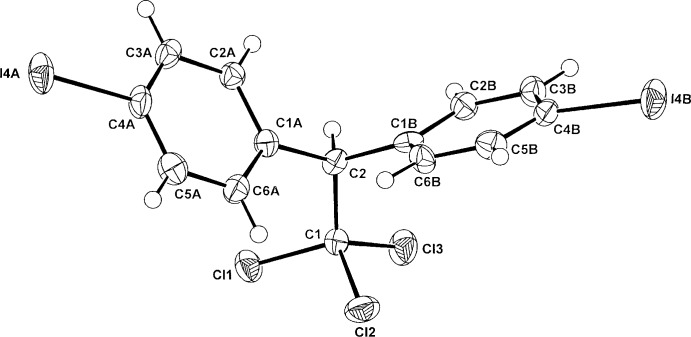
Molecular conformation and atom numbering scheme for the title molecule, with displacement ellipsoids drawn at the 50% probability level.

**Fig. 2.**
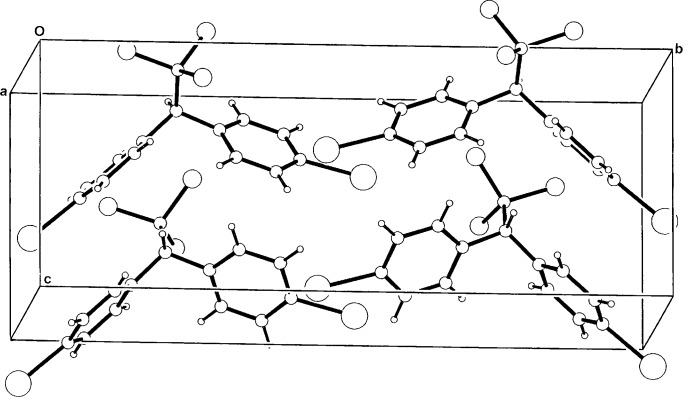
A perspective view of the crystal packing of the title compound viewed along the a axis.

## supplementary crystallographic information

### Comment

The title compound is the *p*-iodophenyl analogue of the insecticide DDT [1,1,1-trichloro-2,2-bis(4-chlorophenyl)ethane] which with the *p*-bromophenyl analogue provided crystal data (Schneider & Fankuchen, 1946) that indicated a probable isomorphous series [orthorhombic, space group *Pca*2_1_, *Z* = 4: for the *p*-Cl analogue (DDT), *a* = 9.963 (1), *b* = 19.200 (2), *c* = 7.887 (1) Å, *V* = 1509.0 Å^3^ [from the crystal structure of DDT (DeLacy & Kennard, 1972); for the 4-bromophenyl analogue, *a* = 9.93, *b* = 19.68, *c* = 7.93 Å, *V* = 1549 Å^3^]. The structure of the title compound, for which the crystal data was also reported by Schneider & Fankuchen (1946), is reported herein.

With the title compound (Fig. 1), isomorphism with DDT as suggested from the crystal data has been confirmed on the basis of space group, cell parameters and the molecular structures. The dihedral angle between the two phenyl planes in this compound [65.8 (4)°] compares with 64.7 (7)° in the structure of DDT (DeLacy & Kennard, 1972). Stabilizing the ring conformation is an intramolecular aromatic C6*A*-H···Cl2 interaction [D···A = 3.335 (10) Å].

In the crystal, there are relatively short I4*A*···Cl1 and I4*A*···I4*A* contacts [3.777 (2) and 4.1502 (9) Å, respectively] but otherwise no other significant intermolecular interactions are present (Fig. 2).

### Experimental

The title compound was obtained as an analytical reference standard from the U.S.Public Health Service. The original crystal data was reported by Schneider & Fankuchen (1946). Small colourless plate-like crystals of the title compound, suitable for X-ray analysis, were obtained by room temperature evaporation of a solution in isopropyl alcohol.

### Refinement

Hydrogen atoms were included in the refinement at calculated positions [C—H = 0.93 Å (aromatic) or 0.98 Å (methine), with *U*_iso_(H) = 1.2*U*_eq_(C), using a riding-model approximation. The maximum difference electron density peak was 0.80 eÅ^-3^, adjacent to atom I4A.

### Crystal data

**Table:**

C_14_H_9_Cl_3_I_2_	*F*(000) = 1000
*M**_r_* = 537.36	*D*_x_ = 2.222 Mg m^−^^3^
Orthorhombic, *P**c**a*2_1_	Mo *K*α radiation, λ = 0.71073 Å
Hall symbol: P 2c -2ac	Cell parameters from 2427 reflections
*a* = 9.8117 (3) Å	θ = 3.2–28.7°
*b* = 20.3445 (4) Å	µ = 4.40 mm^−^^1^
*c* = 8.0486 (2) Å	*T* = 200 K
*V* = 1606.61 (7) Å^3^	Plate, colourless
*Z* = 4	0.25 × 0.20 × 0.08 mm

### Data collection

**Table:**

Oxford Diffraction Gemini-S CCD-detector diffractometer	2905 independent reflections
Radiation source: Enhance (Mo) X-ray source	2687 reflections with *I* > 2σ(*I*)
Graphite monochromator	*R*_int_ = 0.028
Detector resolution: 16.077 pixels mm^-1^	θ_max_ = 26.0°, θ_min_ = 3.4°
ω scans	*h* = −10→12
Absorption correction: multi-scan (*CrysAlis PRO*; Agilent, 2012)	*k* = −13→25
*T*_min_ = 0.386, *T*_max_ = 0.980	*l* = −9→9
5183 measured reflections	

### Refinement

**Table:**

Refinement on *F*^2^	Secondary atom site location: difference Fourier map
Least-squares matrix: full	Hydrogen site location: inferred from neighbouring sites
*R*[*F*^2^ > 2σ(*F*^2^)] = 0.044	H-atom parameters constrained
*wR*(*F*^2^) = 0.112	*w* = 1/[σ^2^(*F*_o_^2^) + (0.0574*P*)^2^ + 5.2653*P*] where *P* = (*F*_o_^2^ + 2*F*_c_^2^)/3
*S* = 1.08	(Δ/σ)_max_ = 0.001
2905 reflections	Δρ_max_ = 0.80 e Å^−^^3^
172 parameters	Δρ_min_ = −0.95 e Å^−^^3^
1 restraint	Absolute structure: Flack (1983), 1203 Friedel pairs
Primary atom site location: structure-invariant direct methods	Flack parameter: −0.02 (4)

### Special details

**Table:**

Geometry. Bond distances, angles *etc*. have been calculated using the rounded fractional coordinates. All su's are estimated from the variances of the (full) variance-covariance matrix. The cell e.s.d.'s are taken into account in the estimation of distances, angles and torsion angles
Refinement. Refinement of *F*^2^ against ALL reflections. The weighted *R*-factor *wR* and goodness of fit *S* are based on *F*^2^, conventional *R*-factors *R* are based on *F*, with *F* set to zero for negative *F*^2^. The threshold expression of *F*^2^ > σ(*F*^2^) is used only for calculating *R*-factors(gt) *etc*. and is not relevant to the choice of reflections for refinement. *R*-factors based on *F*^2^ are statistically about twice as large as those based on *F*, and *R*- factors based on ALL data will be even larger.

### Fractional atomic coordinates and isotropic or equivalent isotropic displacement parameters (Å^2^)

**Table:**

	*x*	*y*	*z*	*U*_iso_*/*U*_eq_	
I4A	0.95323 (7)	0.01062 (3)	1.18735 (8)	0.0400 (2)	
I4B	0.89471 (7)	0.54100 (3)	0.90275 (11)	0.0422 (2)	
Cl1	1.0549 (2)	0.15878 (11)	0.4415 (3)	0.0316 (7)	
Cl2	1.1944 (2)	0.27227 (12)	0.5751 (3)	0.0359 (7)	
Cl3	0.9773 (3)	0.28835 (12)	0.3400 (3)	0.0366 (7)	
C1	1.0324 (8)	0.2408 (4)	0.5112 (12)	0.026 (3)	
C1A	0.9376 (8)	0.1901 (4)	0.7842 (11)	0.022 (3)	
C1B	0.9112 (8)	0.3126 (4)	0.7238 (11)	0.023 (3)	
C2	0.9238 (8)	0.2436 (4)	0.6522 (10)	0.020 (3)	
C2A	0.8197 (9)	0.1581 (4)	0.8344 (11)	0.024 (2)	
C2B	0.9956 (8)	0.3350 (4)	0.8512 (12)	0.027 (3)	
C3A	0.8211 (9)	0.1073 (4)	0.9512 (11)	0.027 (3)	
C3B	0.9885 (8)	0.3989 (4)	0.9066 (15)	0.031 (3)	
C4A	0.9450 (10)	0.0899 (4)	1.0187 (13)	0.030 (3)	
C4B	0.8945 (9)	0.4409 (5)	0.8347 (13)	0.029 (3)	
C5A	1.0643 (9)	0.1209 (5)	0.9744 (13)	0.032 (3)	
C5B	0.8044 (8)	0.4195 (4)	0.7141 (12)	0.029 (3)	
C6A	1.0600 (9)	0.1711 (5)	0.8569 (13)	0.029 (3)	
C6B	0.8130 (8)	0.3553 (4)	0.6603 (12)	0.027 (3)	
H2	0.83640	0.23500	0.59740	0.0250*	
H2A	0.73680	0.17080	0.78850	0.0290*	
H2B	1.05770	0.30630	0.89950	0.0320*	
H3A	0.74120	0.08600	0.98230	0.0320*	
H3B	1.04570	0.41360	0.99090	0.0360*	
H5A	1.14670	0.10850	1.02230	0.0380*	
H5B	0.73940	0.44780	0.67010	0.0350*	
H6A	1.14030	0.19220	0.82650	0.0350*	
H6B	0.75230	0.34020	0.58020	0.0320*	

### Atomic displacement parameters (Å^2^)

**Table:**

	*U*^11^	*U*^22^	*U*^33^	*U*^12^	*U*^13^	*U*^23^
I4A	0.0686 (4)	0.0249 (3)	0.0266 (3)	0.0085 (3)	0.0032 (3)	0.0055 (3)
I4B	0.0629 (4)	0.0206 (3)	0.0430 (4)	−0.0046 (3)	0.0038 (4)	−0.0056 (3)
Cl1	0.0378 (11)	0.0244 (11)	0.0326 (13)	0.0046 (8)	0.0019 (9)	−0.0051 (9)
Cl2	0.0264 (10)	0.0380 (12)	0.0433 (15)	−0.0087 (9)	0.0074 (10)	−0.0060 (11)
Cl3	0.0510 (13)	0.0299 (12)	0.0290 (12)	0.0033 (10)	0.0040 (11)	0.0078 (10)
C1	0.025 (4)	0.019 (4)	0.034 (5)	−0.001 (3)	0.003 (4)	−0.001 (4)
C1A	0.026 (4)	0.020 (4)	0.021 (5)	0.000 (3)	−0.002 (3)	−0.002 (3)
C1B	0.019 (3)	0.031 (5)	0.018 (5)	−0.003 (3)	0.004 (3)	0.000 (3)
C2	0.018 (4)	0.022 (4)	0.021 (5)	−0.004 (3)	−0.007 (3)	0.003 (3)
C2A	0.024 (4)	0.022 (4)	0.026 (4)	−0.001 (3)	−0.001 (4)	−0.001 (3)
C2B	0.020 (4)	0.025 (4)	0.035 (5)	0.005 (3)	−0.008 (4)	0.002 (4)
C3A	0.035 (4)	0.021 (4)	0.024 (5)	−0.007 (3)	0.004 (4)	−0.003 (3)
C3B	0.027 (4)	0.034 (5)	0.031 (5)	−0.001 (3)	−0.003 (4)	−0.002 (5)
C4A	0.044 (5)	0.020 (4)	0.026 (5)	0.003 (4)	0.004 (4)	0.006 (4)
C4B	0.035 (5)	0.020 (4)	0.031 (5)	−0.002 (4)	0.012 (4)	0.001 (4)
C5A	0.033 (5)	0.032 (5)	0.030 (5)	0.005 (4)	−0.005 (4)	0.002 (4)
C5B	0.032 (4)	0.024 (4)	0.030 (6)	0.003 (3)	−0.001 (4)	0.004 (4)
C6A	0.028 (4)	0.024 (4)	0.035 (6)	−0.003 (4)	0.000 (4)	0.003 (4)
C6B	0.025 (4)	0.024 (4)	0.031 (6)	−0.002 (3)	−0.001 (4)	−0.002 (4)

### Geometric parameters (Å, º)

**Table:**

I4A—C4A	2.110 (9)	C3B—C4B	1.384 (13)
I4B—C4B	2.109 (10)	C4A—C5A	1.377 (13)
Cl1—C1	1.774 (9)	C4B—C5B	1.383 (13)
Cl2—C1	1.789 (8)	C5A—C6A	1.393 (15)
Cl3—C1	1.768 (9)	C5B—C6B	1.379 (12)
C1—C2	1.558 (12)	C2—H2	0.9800
C1A—C2	1.527 (12)	C2A—H2A	0.9300
C1A—C2A	1.388 (12)	C2B—H2B	0.9300
C1A—C6A	1.391 (12)	C3A—H3A	0.9300
C1B—C2	1.523 (12)	C3B—H3B	0.9300
C1B—C2B	1.395 (12)	C5A—H5A	0.9300
C1B—C6B	1.394 (12)	C5B—H5B	0.9300
C2A—C3A	1.397 (12)	C6A—H6A	0.9300
C2B—C3B	1.376 (12)	C6B—H6B	0.9300
C3A—C4A	1.378 (13)		
			
Cl1—C1—Cl2	108.5 (4)	C3B—C4B—C5B	121.7 (9)
Cl1—C1—Cl3	107.8 (5)	C4A—C5A—C6A	119.1 (9)
Cl1—C1—C2	110.5 (6)	C4B—C5B—C6B	118.7 (8)
Cl2—C1—Cl3	107.5 (5)	C1A—C6A—C5A	121.1 (8)
Cl2—C1—C2	112.7 (6)	C1B—C6B—C5B	121.2 (8)
Cl3—C1—C2	109.8 (5)	C1—C2—H2	105.00
C2—C1A—C2A	117.6 (7)	C1A—C2—H2	105.00
C2—C1A—C6A	124.6 (8)	C1B—C2—H2	105.00
C2A—C1A—C6A	117.8 (8)	C1A—C2A—H2A	119.00
C2—C1B—C2B	122.1 (7)	C3A—C2A—H2A	119.00
C2—C1B—C6B	119.5 (7)	C1B—C2B—H2B	119.00
C2B—C1B—C6B	118.4 (8)	C3B—C2B—H2B	119.00
C1—C2—C1A	114.8 (7)	C2A—C3A—H3A	121.00
C1—C2—C1B	111.4 (7)	C4A—C3A—H3A	121.00
C1A—C2—C1B	113.6 (7)	C2B—C3B—H3B	121.00
C1A—C2A—C3A	122.3 (8)	C4B—C3B—H3B	121.00
C1B—C2B—C3B	121.1 (8)	C4A—C5A—H5A	120.00
C2A—C3A—C4A	117.7 (8)	C6A—C5A—H5A	120.00
C2B—C3B—C4B	118.8 (9)	C4B—C5B—H5B	121.00
I4A—C4A—C3A	118.9 (7)	C6B—C5B—H5B	121.00
I4A—C4A—C5A	119.0 (7)	C1A—C6A—H6A	120.00
C3A—C4A—C5A	122.0 (9)	C5A—C6A—H6A	119.00
I4B—C4B—C3B	119.1 (7)	C1B—C6B—H6B	119.00
I4B—C4B—C5B	119.1 (7)	C5B—C6B—H6B	119.00
			
Cl1—C1—C2—C1A	44.3 (8)	C6B—C1B—C2—C1A	−134.9 (8)
Cl1—C1—C2—C1B	175.3 (6)	C2—C1B—C2B—C3B	175.7 (8)
Cl2—C1—C2—C1A	−77.2 (8)	C6B—C1B—C2B—C3B	−4.0 (13)
Cl2—C1—C2—C1B	53.8 (8)	C2—C1B—C6B—C5B	−175.6 (8)
Cl3—C1—C2—C1A	163.1 (6)	C2B—C1B—C6B—C5B	4.0 (13)
Cl3—C1—C2—C1B	−65.9 (8)	C1A—C2A—C3A—C4A	0.9 (13)
C2A—C1A—C2—C1	−135.2 (8)	C1B—C2B—C3B—C4B	0.7 (14)
C2A—C1A—C2—C1B	94.9 (9)	C2A—C3A—C4A—I4A	−177.3 (6)
C6A—C1A—C2—C1	44.5 (12)	C2A—C3A—C4A—C5A	0.1 (14)
C6A—C1A—C2—C1B	−85.4 (10)	C2B—C3B—C4B—I4B	−174.4 (7)
C2—C1A—C2A—C3A	178.4 (8)	C2B—C3B—C4B—C5B	2.7 (15)
C6A—C1A—C2A—C3A	−1.4 (13)	I4A—C4A—C5A—C6A	176.9 (7)
C2—C1A—C6A—C5A	−178.8 (9)	C3A—C4A—C5A—C6A	−0.5 (15)
C2A—C1A—C6A—C5A	1.0 (14)	I4B—C4B—C5B—C6B	174.5 (7)
C2B—C1B—C2—C1	−86.2 (10)	C3B—C4B—C5B—C6B	−2.7 (14)
C2B—C1B—C2—C1A	45.5 (11)	C4A—C5A—C6A—C1A	−0.1 (15)
C6B—C1B—C2—C1	93.5 (9)	C4B—C5B—C6B—C1B	−0.8 (13)

### Hydrogen-bond geometry (Å, º)

**Table:**

*D*—H···*A*	*D*—H	H···*A*	*D*···*A*	*D*—H···*A*
C6*A*—H6*A*···Cl2	0.93	2.65	3.335 (10)	131
